# Supplementary material for: Early Life Obesity Increases Neuroinflammation, Amyloid Beta Deposition, and Cognitive Decline in a Mouse Model of Alzheimer’s Disease
Source: Nutrients. 2023 May 27;15(11):2494. doi: 10.3390/nu15112494 (PMC10255742; doi:10.3390/nu15112494)
Supplement: Supplementary file 1 [file nutrients-15-02494-s001.zip › nutrients-2368527-supplementary.pdf]

Supplementary Materials

S1. Results

S1.1. HFD increases non-fasted blood glucose concentrations in both AD and WT mice compared to NC in both sexes.

Blood glucose concentrations were measured prior to termination. There was a significant main effect of diet in both sexes (Females: $F_{1, 37} = 45.62$, $p < 0.0001$; Males: $F_{1, 77} = 31.48$, $p < 0.0001$), and genotype in females (Females: $F_{1, 37} = 4.57$, $p < 0.05$; Males: $F_{1, 77} = 0.73$, $p = 0.3960$) on blood glucose concentrations, with no significant interaction between the two (Females: $F_{1, 37} = 0.007$, $p = 0.9318$; Males: $F_{1, 77} = 2.806$, $p = 0.0980$). Post-hoc tests showed that HFD increased blood glucose concentrations relative to NC-fed mice in all treatment groups ($p < 0.0001$-0.05, Figure S1).

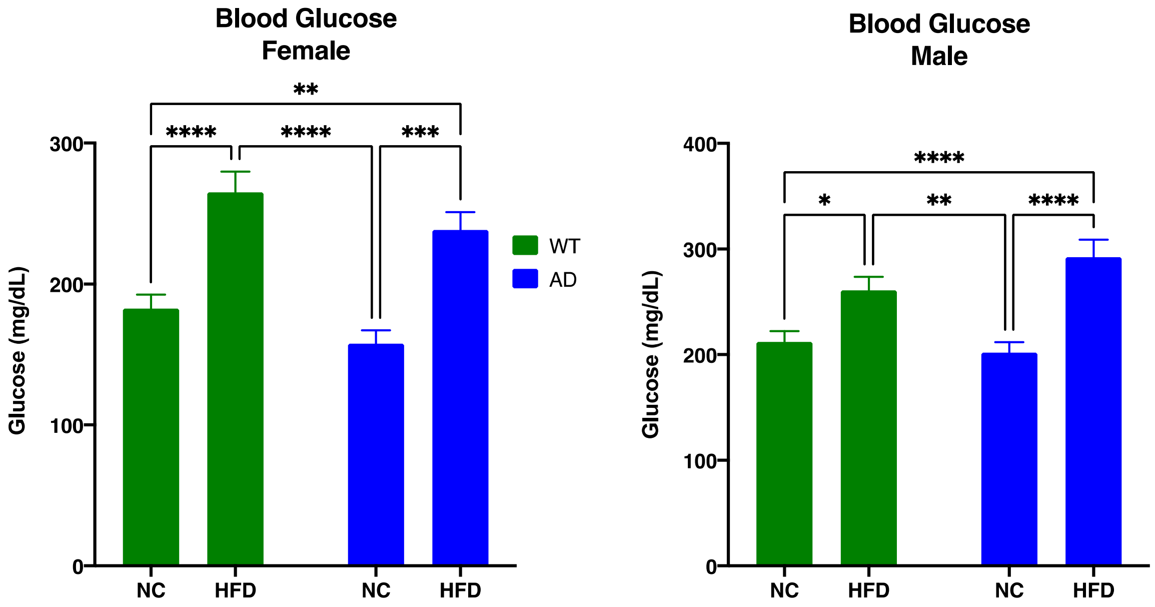

(**A**) (**B**)

Figure S1: HFD increases non-fasted blood glucose in both sexes. (**A**). Non-fasted blood glucose in female mice. (**B**). Non-fasted blood glucose in male mice. Data analyzed via two-way ANOVA and Holm-Sidak's multiple comparisons test. n = 6-12. * p < 0.05, ** p < 0.01, *** p < 0.001, **** p < 0.0001.
